# Clinical and genetic analyses of three Korean families with hereditary hemorrhagic telangiectasia

**DOI:** 10.1186/1471-2350-12-130

**Published:** 2011-10-03

**Authors:** Mi-Jung Kim, Seon-Tae Kim, Hyoung-Doo Lee, Kyu-Yong Lee, Jiyoung Seo, Jae-Bom Lee, Young-Jae Lee, Suk P Oh

**Affiliations:** 1 Lee Gil Ya Cancer and Diabetes Institute, Gachon University, Incheon, Korea; 2 World Class University Program, Gachon University, Incheon, Korea; 3 Department of Otolaryngology, School of Medicine, Gachon University, Incheon, Korea; 4 Department of Pediatrics, Pusan National University College of Medicine, Pusan, Korea; 5 Department of Neurology, Hanyang University College of Medicine, Seoul, Korea; 6 Department of Life Science, Hallym University, Chuncheon, Korea; 7 Department of Physiology and Functional Genomics, College of Medicine, University of Florida, Gainesville, FL, USA

## Abstract

**Background:**

Hereditary hemorrhagic telangiectasia (HHT) is an autosomal-dominant vascular disorder, characterized by recurrent epistaxis, mucocutaneous telangiectases, and arteriovenous malformations (AVMs) in various visceral organs. Endoglin (*ENG*) and activin receptor-like kinase 1 (*ACVRL1; ALK1*), receptors for transforming growth factor-β (TGF-β) superfamily, have been identified as the principal HHT-causing genes.

**Methods:**

Three unrelated Korean HHT patients and their asymptomatic as well as symptomatic family members were genetically diagnosed by sequencing whole exons and their flanking regions of *ENG *and *ACVRL1*. Functionality of an aberrant translation start codon, which is created by a substitution mutation at the 5'-untranslated region (UTR) of *ENG *found in a HHT family, was tested by transient *in vitro *transfection assay. Decay of the mutant transcripts was also assessed by allele-specific expression analysis.

**Results:**

Two *ENG *and one *ACVRL1 *mutations were identified: a known *ENG *mutation (c.360+1G > A; p.Gly74_Tyr120del); a novel *ENG *mutation (c.1-127C > T); and a novel *ACVRL1 *mutation (c.252_253insC; p.Val85fsX168). We further validated that the 5'-UTR *ENG *mutation prevents translation of ENG from the biological translation initiation site of the mutant allele, and leads to degradation of the mutant transcripts.

**Conclusions:**

This is the first experimental demonstration that a 5'-UTR mutation can prevent translation of ENG among HHT patients, and further supports the previous notion that haploinsufficiency is the primary mechanism of HHT1. Our data also underscore the importance of including exons encoding 5' UTR for HHT mutation screening.

## Background

Hereditary hemorrhagic telangiectasia (HHT) is a rare genetic vascular disorder, inherited in an autosomal-dominant trait. With a regional variance, HHT affects about 1 in 5,000 to 8,000 people worldwide [1-3]. Major clinical diagnosis criteria include family history, recurrent epistaxis, mucocutaneous telangiectases, and arteriovenous malformations (AVMs) in internal organs such as the lung, brain, and liver [4,5]. Epistaxis is usually the earliest clinical symptom of HHT that appears at teens, while cutaneous and gastrointestinal telangiectases manifest at adulthood ages. However, both of these symptoms progressively worsen with age, and manifest in more than 90% of HHT patients in their 60's [1].

Five clinically indistinguishable types of HHT are defined by associated genetic loci. HHT1 (MIM #187300) and HHT2 (MIM #600376) that account for more than 80% of HHT are caused by heterozygous mutations in endoglin (*ENG*) [6-8] and activin receptor-like kinase 1 (*ACVRL1; ALK1*) [9-11] genes, respectively [12,13]. To date, 397 *ENG *and 332 *ACVRL1 *mutations and polymorphisms have been reported [14]. Two additional genetic loci on chromosomes 5 and 7 were identified for HHT3 (MIM #601011) and HHT4 (MIM #610655), respectively [15,16]. A group of juvenile polyposis patients harboring mutations in the *SMAD4 *gene display a combined syndrome with HHT (JP-HHT) (MIM #175050) [17]. Based on the fact that ENG, ALK1, and SMAD4 are receptors or intracellular mediator of transforming growth factor-β (TGF-β) family signals, it has been postulated that impaired signaling of TGF-β family member(s) would be an important pathological mechanism of HHT. Recent biochemical studies have suggested that BMP9 is the plausible physiological ligand of ALK1 [18-20].

Since clinical diagnosis of HHT has limitations due to the late onset, incomplete penetrance, and variability of disease manifestations, genetic diagnosis is valuable not only to confirm the clinical diagnosis but also to identify asymptomatic carriers among a HHT family. Early screening of asymptomatic patients for cerebral and pulmonary AVMs that are found in about 40% of HHT patients can prevent them from potential serious health risks such as stroke and brain abscess [1].

Despite dozens of HHT case reports for Korean HHT families, only one genetic study identifying genetic mutations has been reported [21]. Here, we report genetic analyses of three unrelated Korean HHT families diagnosed by clinical criteria. We found one known and one novel mutations in *ENG*, and one novel mutation in *ACVRL1*. With molecular analysis, we demonstrate that the novel *ENG *mutation found in the 5'-untranslated region (UTR) initiates aberrant translation that prevents translation of ENG from the biological translation initiation site of the mutant allele, and leads to nonsense-mediated mRNA decay.

## Methods

### Human subjects

Experimental procedures performed were reviewed and approved by the Ethics Committee, an institutional review board, at Lee Gil Ya Cancer and Diabetes Institute, Gachon University, Incheon, Korea. Patients were diagnosed with HHT when they possessed at least three of the four Curaçao established criteria: 1) an affected first degree family member; 2) recurrent nosebleeds; 3) multiple telangiectases along the mucocutaneous surface; and 4) AVMs in major organs [22]. HHT genetic analysis was then conducted after obtaining informed consent from patients and their family members. Clinical features of patients reported by clinicians are summarized in Table 1. Only obviously detected symptoms are listed. Since systematic screening was not employed, visceral AVMs and skin telangiectasia might have been missed from some patients.

**Table 1:** Clinical features and mutations of three HHT families

Family	Patient	Gender/Age	Clinical features	Mutations
**I**	I-1	M/46	Epistaxis	*ENG *c.360+1G > A, p.Gly74_Tyr120del
	I-2	M/43	Epistaxis, PAVM, Epilepsy, Cerebral abscess	*ENG *c.360+1G > A, p.Gly74_Tyr120del
	I-3	F/41	Epistaxis, PAVM	*ENG *c.360+1G > A, p.Gly74_Tyr120del
	I-4	F/14	Epistaxis, PAVM	*ENG *c.360+1G > A, p.Gly74_Tyr120del
	I-5	F/12	Epistaxis	*ENG *c.360+1G > A, p.Gly74_Tyr120del
**II**	II-1	F/82	Epistaxis, Skin telangiectasia	*ENG *c.1-127 C > T, new upstream translation start codon (TSC)
	II-2	M/65	Epistaxis, CAVM	*ENG *c.1-127 C > T, new upstream TSC
	II-3	M/53	Epistaxis	*ENG *c.1-127 C > T, new upstream TSC
	II-4	F/50	Epistaxis	*ENG *c.1-127 C > T, new upstream TSC
	II-5	M/13	Epistaxis, CAVM, dead	n/d
	II-6	M/18	Epistaxis, PAVM, Seizure, Embolic cerebral infarction	*ENG *c.1-127 C > T, new upstream TSC
**III**	III-1	M/55	Epistaxis	*ACVRL1 *c.252_253insC, p.Val85fsX168
	III-2	M/50	Epistaxis	*ACVRL1 *c.252_253insC, p.Val85fsX168
	III-3	F/40	Epistaxis	*ACVRL1 *c.252_253insC, p.Val85fsX168

n/d: not determined

### Genomic DNA isolation

Blood samples were collected in vacuum blood collection tubes containing EDTA. Genomic DNA was isolated from 200-μl blood samples using the Exgene Blood SV mini kit (GeneAll, South Korea) according to the manufacturer's protocol.

### PCR amplification and sequence analysis of *ENG *and *ACVRL1 *exons

All exons of *ENG *and *ACVRL1 *genes, including non-coding regions and flanking intronic sequences, were amplified by genomic polymerase chain reaction (PCR) using PrimeSTAR HS DNA Polymerase (TAKARA, Japan) and the appropriate primer sets (Additional file 1; Table S1). PCR products were separated in a 1-2% agarose gel and purified using QIAquick Gel Extraction Kit (QIAGEN, Germany) or Expin PCR SV kit (GeneAll, Korea). These purified PCR products were then directly sequenced. Accession numbers of the reference sequences used in this study are [GenBank:NM_000118.2] (cDNA) and [GenBank:NC_009551.1] (genomic DNA) for *ENG*, and [GenBank:NM_000020.2] (cDNA) and [GenBank:NC_009545.1] (genomic DNA) for *ACVRL1*.

### Plasmid construction and transient in vitro transfection

**Figure 1:**
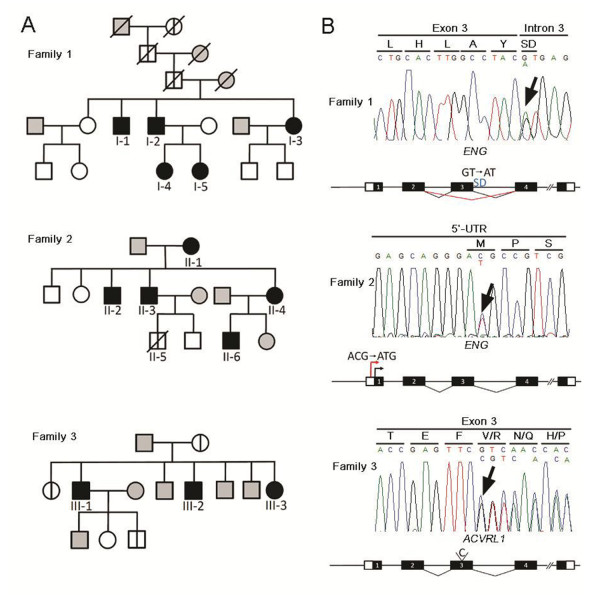
**Pedigree and genetic analysis of three HHT families**. (**A**) Pedigree of families with genetic mutations and/or symptoms of HHT. Pedigree symbols: filled symbol, affected individual; open symbol, unaffected individual; divided symbol, affected individual by hearsay; gray symbol, no phenotypical and genetic data available. A slashed symbol indicates that the individual is deceased. Genetic analysis was performed on all individuals represented by filled and open symbols. Genetic mutations and HHT-associated symptoms of numbered patients are summarized in Table 1. (**B**) Genetic studies of three representative family members with HHT. Family 1, *ENG *c.360+1G > A (p.Gly74_Tyr120del); Family 2, *ENG *c.1-127 C > T (aberrant translation); Family 3, *ACVRL1 *c.252_253insC (p.Val85fsX168). The amino acid translation is shown above each codon. The amino acid sequence in Family 2 is the predicted sequence translated from the aberrant translation start codon. Arrows indicate mutation sites. SD, splicing donor sequence.

**Figure 2:**
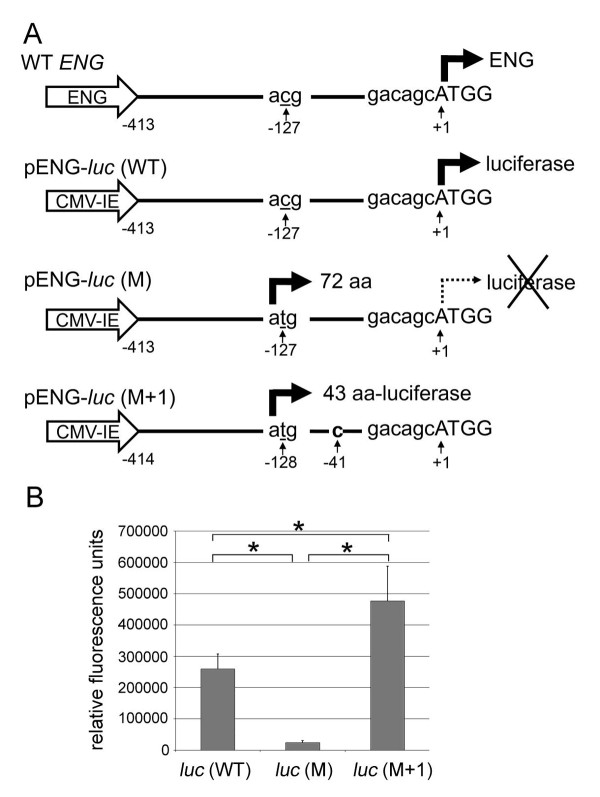
**Functionality of the aberrant translation start codon**. (**A**) Schematic representation of the pENG-*luc *constructs. Wild-type (WT) *ENG *promoter produces *ENG *mRNA including the 413-nt-5'-UTR. pENG-*luc*(WT) was designed to code for *luciferase *mRNA containing the same *ENG *5'-UTR under the control of the immediate early promoter of CMV (CMV-IE). In pENG-*luc*(M), the 'C' at position -127 in pENG-*luc*(WT) was replaced with 'T' (underlined), which generated the aberrant translation start codon. The substitution was found in the mutant allele of HHT patients in Family 2. The aberrant translation start codon putatively produces a 72-amino acid-protein out of frame with the *luciferase *coding sequence. In pENG-*luc*(M+1), a 'C' nucleotide (bold) was inserted between -41 and -40 of pENG-*luc*(M), thus producing an aberrant translation start codon that was in frame with the *luciferase *coding sequence. Nucleotides are numbered with c.1 corresponding to the 'A' of the ATG translation start codon in the reference sequence [GenBank:NM_000118.2]. (**B**) Bar graph illustrating the results of luciferase assays. HepG2 cells were transfected with pENG-*luc *plasmids [pENG-*luc*(WT), pENG-*luc*(M), or pENG-*luc*(M+1)] plus the β-galactosidase plasmid. Luciferase activity was normalized to β-galactosidase activity. The luciferase activity of pENG-*luc*(M) was significantly reduced compared to that of pENG-*luc*(WT). The activity was restored to levels even higher than WT levels in pENG-*luc*(M+1), which produced an in-frame luciferase fusion protein (44 aa-luciferase). Separate transfections were performed for each of three separate experiments (n = 9). Data represent mean ± SD. *p < .0001, as determined by Student's *t *test.

*ENG *5'-UTRs (position -413 to -1) of wild-type (WT) and mutant (M) alleles from genomic DNA of HHT patients in Family 2 (Figures 1) were amplified via PCR and subcloned into T&A Cloning Vectors (Real Biotech Corporation, Taiwan), yielding pTA-ENG-WT and pTA-ENG-M. The *ENG *c.1-127 C > T mutation created a putative translation start codon in the 5'-UTR of pTA-ENG-M. WT and mutant 5'-UTRs were amplified with hENF-5UTR-F1 (gctagcctctacccggttggcaggcggcct) and hENF-5UTR-R1 (ccatgGtgtccacgtgggggcctgtgcg) from WT and mutant *ENG *alleles, respectively. For cloning purpose, an NheI site (underlined) was introduced at the 5'-end of the forward primer and the 'G' nucleotide at position -2 in the 5'-UTR was replaced with 'C', which produced an NcoI site (underlined) at the 5'-end of the reverse primer. The *luciferase *gene in pGL4.14 [luc2/hygro] (Promega, USA) was subcloned into the NcoI and XbaI sites in pTA-ENG-WT and pTA-ENG-M plasmids, generating pTA-ENG-WT-luc and pTA-ENG-M-luc, respectively. The eGFP gene in pEGFP-N1 (Clontech, USA) was replaced with the *ENG *5'-UTR-luciferase fragments of pTA-ENG-WT-luc and pTA-ENG-M-luc. The substituted 'C' nucleotide at position -2 to produce the NcoI site reverted to the original 'G' nucleotide by site directed mutagenesis (COSMO GENETECH, Korea), generating pENG-*luc*(WT) and pENG-*luc*(M), respectively (Figure 2A). A plasmid with an artificial 5'-UTR, which was produced by insertion of a 'C' nucleotide between -41 and -40 of the mutant 5'-UTR in pENG-*luc*(M), was generated and designated pENG-*luc*(M+1) (Figure 2A). The inserted 'C' nucleotide between -41 and -40 resulted in a frameshift and produced a luciferase fusion protein translated from the aberrant translation start codon (Figure 2A).

The plasmids were transfected into HepG2, a human hepatocellular liver carcinoma cell line, with Magnetamin (LPS Solution, South Korea) according to manufacturer's instructions. Cells were harvested 48 h after transfection. The luciferase activity was measured with the Enhanced Luciferase Assay Kit (BD Biosciences, USA) and normalized with β-galactosidase activity using β-galactosidase Enzyme Assay System (Promega, USA).

### Allele-specific expression analysis

Total RNAs were isolated from blood using RNAiso Plus (TAKARA, Japan). Reverse transcription was performed with Prime RT-Premix (2×) (Genet Bio, Korea) using *ENG*-specific R3 primer (5'-cctggagagtcagctccagctgtg-3'). The PCR primers, F2 (5'-ctgctgtcactgccatccattgga-3') and R2 (5'-agacctggctagtggtatatgtca-3'), were used for amplification of the *ENG *cDNA. Genomic DNA was amplified using F1 (5'-ccatccttcggacagcaactccag-3') and R1 (5'-ccaccctgggtccctggacaccta-3'). Amplified PCR products were digested with BtsCI, which recognizes GGATG (i.e. mutant specific) sequence, and analyzed on an 8% polyacrylamide gel to determine whether the PCR product possessed *ENG *c.1-127 C > T mutation. The PCR products were also subjected to direct sequencing.

### Real-time RT-PCR

Total RNAs were isolated from blood samples of unaffected and affected individuals using RNAiso Plus (TAKARA, Japan) and first-strand cDNA was synthesized by PrimeScript RT Master Mix Kit (TAKARA, Japan) with oligo(dT) primer. Real-time PCR was carried out using SYBR Premix DimerEraser Kit (TAKARA, Japan) and CFX384 Real-Time system (Bio-Rad, USA). A human Cyclophilin primer set (Forward, 5'-tgccatcgccaaggagtag-3'; Reverse, 5'-tgcacagacggtcactcaaa-3') was used to normalize the amount of total cDNA in each sample. Predesigned PCR primer for amplifying the Exon 3 and 4 region of the *ENG *gene (PrimerBank ID 168693646b1) [23-25] was used to determine the amount of *ENG *cDNA in each sample. Each sample was run in triplicate. After 40 cycles of PCR reaction, the relative amount of *ENG *transcripts was determined using the ΔΔCt method.

## Results

### Clinical features and mutations

Clinical features and mutations of patients are summarized in Table 1. Pedigree of HHT families is represented in Figure 1A.

#### Family 1

**Figure 3:**
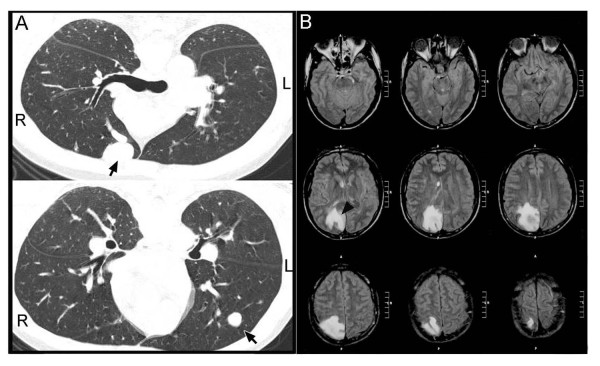
**Clinical features of an HHT patient (I-2)**. PAVM (arrows) and cerebral abscess (arrow head) are detected by chest computed tomography (CT) (A) and brain CT (B), respectively.

The proband is a 43-year-old man (I-2), diagnosed as HHT with recurrent epistaxis, pulmonary AVM (PAVM) (Figure 3A), and family history of epistaxis and/or PAVM. He had also suffered for epilepsy and cerebral abscess (Figure 3B) when he was 26 and 40 years old, respectively. These brain symptoms were most likely due to PAVMs that were recently treated with coil embolization. His two daughters (I-4 and I-5), brother (I-1), and sister (I-3), have experienced frequent nosebleeds and/or PAVM. This trait can be traced to great-grandmother of the proband.

#### Family 2

The proband is an 18-year-old man (II-6). He was diagnosed as HHT with recurrent epistaxis, PAVM, and family history of recurrent epistaxis [26]. He had seizure and embolic cerebral infarction [26]. These neurological complications might be associated with PAVM. Stroke, cerebral abscess, seizure, transient ischemic attacks, and migraine headaches were observed in 9%, 8%, 37%, and 43% PAVM patients, respectively [27]. A history of epistaxis was found in his relatives on the mother's side (patient II-1, II-2, II-3, II-4) [26]. His grandmother (II-1) has telangiectasis lesions on the lips and skin, and his uncle (II-2) had cerebral AVM (CAVM), and his cousin (II-5) died of CAVM.

#### Family 3

The proband is a 55-year-old man (III-1) who was suspected to be a HHT patient due to recurrent epistaxis and familial history. His brother (III-2) and sister (III-3) suffer from epistaxis. No history of visceral AVM was reported in the family.

### Genetic analysis

PCR amplicons of whole exons and their flanking regions of *ENG *and *ACVRL1 *from genomic DNA of HHT patients and their family members (Figure 1, Table 1) were directly sequenced.

#### Family 1

We identified a heterozygous mutation in the splicing donor sequence in intron 3 of *ENG *(c.360+1G > A) from the proband sample. This mutation was previously reported that it causes an in-frame deletion of exon 3 (47 amino acids; p.Gly74_Tyr120del) and that the mutant protein fails to localize at the plasma membrane but stays in the cytosol [28]. Genetic analysis from symptomatic and asymptomatic family members identified the same mutation from family members with epistaxis and/or PAVM. No mutation was found in two children of the affected proband's sister (I-3).

#### Family 2

No mutation was found in the coding region and splicing junctions of either of *ENG *and *ACVRL1 *in the proband (II-6), but a substitution mutation in the 5'-UTR of *ENG *c.1-127 C > T. Interestingly, the same mutation was found in his mother (II-4, obligate carrier) and only in her symptomatic family members (II-1, -2, -3). The suspected mutation (-128A***C***G-126 with -128A***T***G-126) could generate a putative translation start codon in the 5'-UTR. If the putative translation start codon is functional, the *ENG *mutant allele would prevent the translation initiation from the translation start codon of normal ENG protein and produce an 84-amino acid-protein out of frame with the *ENG*-coding sequence instead. We confirmed with molecular analyses (described below) that this is indeed the case.

#### Family 3

We identified a novel 1-base pair (bp) insertional mutation in the *ACVRL1 *locus from the proband (III-1), and the same mutation was found in his affected sibling (III-2, -3). The 'C' nucleotide insertion in exon3 (c.252_253insC) would result in premature termination of translation (p.Val85fsX168) and production of truncated protein of size 167 amino acids instead of 503 amino acids full-length protein.

### Functional tests of aberrant translation start codon found in Family 2

To investigate the extent to which the putative translation start codon created by mutation in the 5'-UTR of *ENG *identified in Family 2 inhibits normal ENG translation, we generated luciferase reporter constructs. These reporter constructs contain 5'-UTR regions (-413 to -1) of wild-type (WT) or of the mutant (M; c.1-127 C > T) *ENG *gene including the biological translation initiation sequence that was fused in frame with luciferase cDNA (Figure 2A). If the putative translation start codon by c.1-127 C > T mutation was functional and dominant over the endogenous start codon, the translated product from the putative start codon would be out of frame with the *luciferase *coding sequence, and thus no luciferase activity would be detected. As shown in Figure 2B, the luciferase activity from the cells transfected with pENG-*luc*(M) was dramatically lower than that with pENG-*luc*(WT), suggesting that the putative translation initiation sequence functions as the dominant translation start codon and prevents translation of functional luciferase. This notion was further supported by the results from the assay using the pENG-*luc*(M+1) reporter, in which single nucleotide was inserted between positions -41 and -40 of the pENG-*luc*(M). This single nucleotide insertion makes the translation from the putative translation start codon of pENG-*luc*(M+1) reporter in frame with the *luciferase *coding sequence. The luciferase activity from the cells transfected with the pENG-*luc*(M+1) reporter was even higher than that with the pENG-*luc*(WT) reporter, indicating that the putative translation start codon was able to produce a functional luciferase fusion protein. These results demonstrate that the aberrant translation start codon in the mutant *ENG *allele of Family 2 is functional and inhibits normal translation of the endogenous ENG protein.

**Figure 4:**
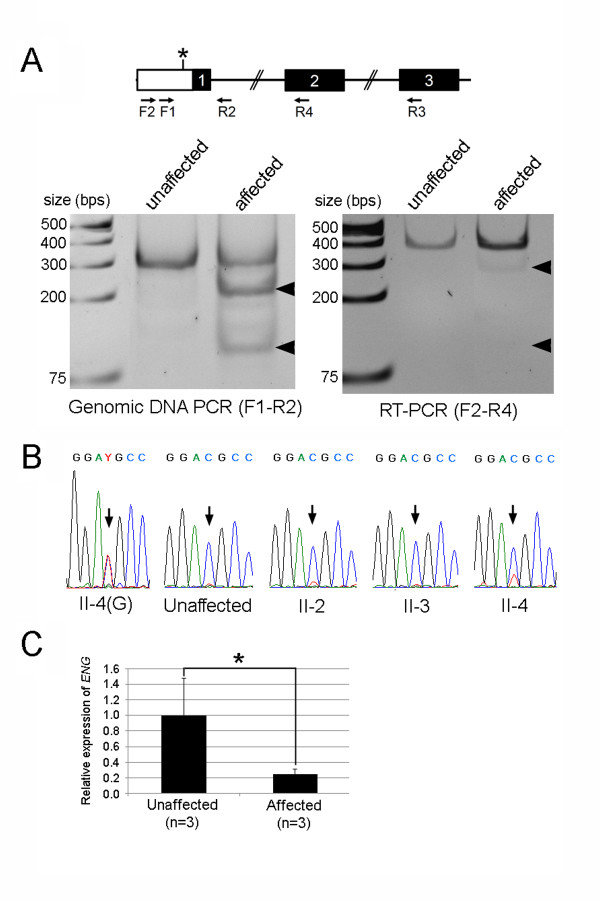
**Allele-specific expression analyses of the *ENG *c.1-127 C > T mutant allele**. (**A**) Restriction fragment length polymorphism analysis. (**Left panel**) F1 and R1 primers were used for amplification of the *ENG *genomic DNA. BtsCI, which recognizes the mutant allele, digested of 313 bp PCR product from the mutant allele into 216 bp and 97 bp fragments (arrowheads). The mutant allele-specific BtsCI-digested PCR products were detected in affected family member (Patient II-3) but not in an unaffected family member. (**Right panel**) F2 and R2 primers were used to amplify ENG transcripts from *ENG *cDNA generated by reverse transcriptase using *ENG*-specific R3 primer. BtsCI digestion of RT-PCR product amplified from mutant allele is supposed to yield 284 bp and 97 bp fragments if the mutant transcripts are stably present. Unlike genomic PCR products, BtsCI-digested PCR fragments from RT-PCR products of affected individuals were barely detectable (arrowheads). (**B**) Direct sequencing of the PCR products amplified from genomic DNA of a patient II-4 and cDNAs of an unaffected family member and three patients (II-2, II-3, II-4). The *ENG *c.1-127 positions were indicated by arrows. (**C**) Real-time RT-PCR analysis of *ENG *transcripts. Total RNAs isolated from the blood of three unaffected persons and three patients (II-2, II-3, II-4) were used for first-strand cDNA synthesis. Mean values of *ENG *ΔΔCt ratios and standard deviations are shown as filled box and bar above each box, respectively. The relative amount of *ENG *transcripts in each sample was normalized with the amount of Cyclophilin in the sample. * *p *< 0.01, compared with the unaffected samples, as determined by Student's *t *test.

The aberrant translation start codon in the mutant allele creates a new open reading frame (ORF), which is 252 bp in length and has a termination codon in exon2. The mutant transcript could be a target of the nonsense-mediated mRNA decay [29,30], a surveillance mechanism, which degrades transcripts with nonsense mutations for preventing the production of erroneous proteins. To test this, we performed a restriction fragment length polymorphism (RFLP) analysis and direct sequencing of genomic DNA- and RT-PCR products using templates isolated from blood samples of unaffected and affected family members. The *ENG *c.1-127 C > T mutation introduces the BtsCI restriction site (GGATG) on the PCR product amplified from the mutant allele. As anticipated, BtsCI digestion on PCR products from genomic DNA templates demonstrated the presence of PCR amplicon of the mutant allele only from affected individuals (Figure 4A). On the other hand, the BtsCI-sensitive PCR amplicon was barely detectable from RT-PCR templates of affected individuals (Figure 4A), suggesting a much lower level of transcripts from the mutant allele compared with those from WT allele. Direct sequencing of RT-PCR products also confirmed the diminished mutant transcripts. Direct sequencing of PCR amplicons from genomic template of an affected family member (patient II-4) showed similar height of 'C' and 'T' peaks amplified from WT and mutant alleles, respectively (Figure 4B). However, the mutant 'T' peak was hardly detected in direct sequencing of RT-PCR products of three patients (patient II-2, -3, -4). To further confirm nonsense-mediated mRNA decay of *ENG *transcripts from the mutant allele, we quantified the amount of *ENG *mRNA using a real-time RT-PCR on RNAs isolated from unaffected and affected individuals (Figure 4C). The levels of *ENG *transcripts in the affected members (II-2, II-3, II-4) appear to be significantly lower than those in unaffected controls. Taken together, these results suggest that the substitution mutation (*ENG *c.1-127 C > T) in the 5'-UTR of *ENG *creates an aberrant translation start site which leads to frameshift and nonsense-mediated mRNA decay of the mutant transcript.

## Discussion

We identified two *ENG *and one *ACVRL1 *mutations in genetic analyses of three unrelated Korean HHT families. Here, the *ENG *c.1-127 C > T mutation in Family 2 and the *ACVRL1 *c.252_253insC mutation in Family 3 are reported for the first time (Figure 1). Through *in vitro *luciferase reporter assay, we demonstrated that the C > T substitution (c.1-127 C > T) in the 5'-UTR of *ENG *is the disease-causing mutation in Family 2 (Figure 1, 2). Mutations in the 5'-UTR are very rare in HHT patients. Bossler *et al*. reported the c.1-10 C > T mutation in the *ENG *5'-UTR of a patient with HHT [31], which is the only known 5'-UTR mutation among reported *ACVRL1 *and *ENG *mutations, except large deletion mutations that include the 5'-UTRs [14]. Although the patient with the *ENG *c.1-10 C > T mutation had recurrent epistaxis, PAVM, and a suggestive family history, no experimental data testing the functionality of the mutant allele was reported.

Generally, eukaryotic ribosomes bind to 5'-end cap of mRNA and scan along the mRNA in the 5' to 3' direction until the first translation start codon is found, thus initiating protein synthesis [32]. This 'position effect' is evident in cases in which a mutation creates an ATG codon upstream of the endogenous translation start codon [32]. The new ATG initiates translation and suppresses translation from the endogenous ATG. The optimal sequence surrounding the translation start codon is 5'-gcc(a/g)ccATGg-3' in mammals [32]. Within the context, the purine (A or G) at position -3 is the most important nucleotide. Additionally, the 'G' at position +4 is also highly conserved, especially in the absence of 'A' at position -3. According to mutagenesis experiments, the sequence contexts generating the most robust translation are 5'-annATGn-3' or 5'-gnnATGg [32]. When the context lacks both the purine at position -3 and the 'G' at position +4, most ribosomes skip the ATG and continue scanning the sequence downstream. In the case of a sequence in which only one of these nucleotides is conserved, initiation of translation from the suboptimal ATG depends on several factors including downstream secondary structure and the remaining nucleotides surrounding the ATG codon [32]. The endogenous and mutant translation start codons in the mutant *ENG *allele are of the following sequence contexts: 5'-gacagcATGg-3' (endogenous) and 5'-gcagggATGc-3' (mutant). The sequences surrounding the endogenous translation start codon contains a purine (A) at position -3 and 'G' at position +4. On the other hand, the mutant translation start context has a purine (G) at position -3, but 'C' instead of 'G' at position +4. Our data from the functional assays reported here demonstrate that the suboptimal mutant translation start context is functionally active and dominate over the translation from the endogenous translation start context (Figure 2). Furthermore, we present that the mutation results in mRNA degradation (Figure 4), which might be caused by nonsense-mediated mRNA decay [29,30]. A critical determinant of nonsense-mediated mRNA decay is the distance between the translation start codon and premature termination codon. Generally, premature termination codons located at least 50-55 nt upstream of the last exon-exon junction leads to strong nonsense-mediated mRNA decay responses. The *ENG *c.1-127 C > T mutation creates a new open reading frame (ORF), which is 252 bp in length. The distance between the termination codon of the new ORF and the last exon-exon junction is more than 1.5 kb, which satisfies the 50-55 nt boundary rule.

## Conclusions

Three unrelated Korean HHT families were analyzed clinically and genetically. We found HHT-causing *ENG *or *ACVRL1 *mutations in these families. Consistent with HHT clinical reports worldwide, the mutation carriers exhibit diverse clinical symptoms even among family members, and epistaxis is the most common symptom among HHT1 and HHT2 patients. We presented experimental demonstration for the first time in the HHT field that a 5'-UTR mutation creates alternative translation initiation sequence, suppresses normal translation of ENG from its endogenous translation start codon, and leads to degradation of the mutant transcripts. In addition to other known cases [4,33,34], this finding provides further supporting evidence that haploinsufficiency is predominant mechanism of HHT1. Our data also underscore the importance of including exons encoding 5' UTR for HHT mutation screening.

## Competing interests

The authors declare that they have no competing interests.

## Authors' contributions

YJL and SPO were the principal investigators and take primary responsibility for the paper. S-TK, HDL, and KYL recruited the patients and performed clinical diagnosis and treatment. M-JK, JS, and J-BL performed the mutational analysis. YJL, SPO, and M-JK co-ordinated the research. YJL and SPO wrote the paper. All authors read and approved the final manuscript.

## Pre-publication history

The pre-publication history for this paper can be accessed here:

http://www.biomedcentral.com/1471-2350/12/130/prepub

## Supplementary Material

Additional file 1 **A title listing the PCR primers for ACVRL1 and ENG genomic PCR**. PCR primer sequences that were used for amplifying exons and their flanking intronic sequences for sequencing *ACVRL1 *and *ENG *genes. Click here for file
