# Supplementary material for: Clinical and genetic analyses of three Korean families with hereditary hemorrhagic telangiectasia
Source: BMC Med Genet. 2011 Oct 3;12:130. doi: 10.1186/1471-2350-12-130 (PMC3202234; doi:10.1186/1471-2350-12-130)
Supplement: Additional file 1 — A title listing the PCR primers for ACVRL1 and ENG genomic PCR. PCR primer sequences that were used for amplifying exons and their flanking intronic sequences for sequencing ACVRL1 and ENG genes. [file 1471-2350-12-130-S1.DOCX]

**Additional files**

**Additional file 1**

**Table S1. PCR primers for *ACVRL1* and *ENG* genomic PCR.**

| ALK1 | Forward  Primer | Sequence | Reverse  Primer | Sequence | Product  Size (bp) |
| --- | --- | --- | --- | --- | --- |
| Ex1 | ALK1e1F | gggtgggtcccggtcctg | ALK1e1R | ccgtccgcggacctcgac | 366 |
| Ex2 | ALK1e2F | ctctgtgatttcctctgggca | ALK1e2R | tacattctccccagcttctcaa | 276 |
| Ex3 | ALK1e3F | ctgggaccacagtggctgagct | ALK1e3R | tttattggccagagcatgagagga | 379 |
| Ex4 | ALK1e4F | gagctgacctagtggaagctga | ALK1e4R | tctgattctgcagttcctatctg | 320 |
| Ex5 | ALK1e5F | aggagcttgcagtgacccagca | ALK1e5R | atgagagcccttggtcctcatcca | 242 |
| Ex6 | ALK1e6F | gaggcagcgcagcatcaagat | ALK1e6R | caaacttgagccctgagtgcag | 298 |
| Ex7 | ALK1e7F | tgacgactccagcctcccttag | ALK1e7R | caagctccgcccacctgtgaa | 389 |
| Ex8 | ALK1e8F2 | tccgtgcacgtctccatctgcctt | ALK1e8R | ggctccacaggctgattcccctt | 373 |
| Ex9 | ALK1e9F | tcctctgggtggtattgggcctc | ALK1e9R | tcagtatctatgagatgaagcaga | 305 |
| Ex10 | ALK1e10F | tctcctctgcacctctctcccaa | ALK1e10R | actcacactacctctacccagata | 256 |
|  |  |  |  |  |  |
| ENG |  |  |  |  |  |
| Ex1 | ENGe1F | cacccagtgacaaagcccgtggca | ENGe1R | cctggtccgtgcaccggaggccga | 563 |
| Ex2 | ENGe2F | cctcataaggtggctgtgatgatg | ENGe2R | catctgccttggagcttcctct | 340 |
| Ex3 | ENGe3F | ctgcctgtctgggtggcacaacct | ENGe3R | cagtagggacctcccatggccaga | 269 |
| Ex4 | ENGe4F | atacttcctgacctcctacatgg | ENGe4R | cctgcactcttggtgcccaagttt | 342 |
| Ex5 | ENGe5F | gggctctgttaggtgcagggct | ENGe5R | tggggtggggctttataaggga | 325 |
| Ex6 | ENGe6F | gcctgtccgcttcagtgttccatc | ENGe6R | ggaaacttccctgatccagaggtt | 236 |
| Ex7 | ENGe7F | gaccgaggcctggcataaccct | ENGe7R | cagtgtggccactgatccaagg | 323 |
| Ex8 | ENGe8F | atcacacagtgaccagccgcct | ENGe8R | ggctaggggaggaaccagatgtc | 249 |
| Ex9-10 | ENGe9a-9bF | gtgtggcaggccacagagacccca | ENGe9a-9bR2 | attccagacacacatggcttgcca | 598 |
| Ex11 | ENGe10F2 | tcctggctggcgccgccagattga | ENGe10R | caggctgtctccctcctgactct | 287 |
| Ex12 | ENGe11F | ttctttccactgtgaggactcag | ENGe11R2 | cttcctgcaaaccacagacctgga | 444 |
| Ex13 | ENGe12F | cagggagtaaacctggaagccgcct | ENGe12R | tcagccactagaacaaacccgaga | 255 |
| Ex14 | ENGe13F | gcagagtggcagtgctgatggcgt | ENGe13R | tgctgctctccgaggaggccg | 347 |
| Ex14 | ENGe14F | aggaccctgacctccgcc | ENGe14R | cactggcagcaggcctctgaga | 328 |
| Ex14 | ENG3UTRF | ctcgacccagaatggagcctgct | ENG3UTRR | ccaccctggctttgaatgtcactg | 624 |
